# Use of the Er:YAG Laser in Conservative Dentistry: Evaluation of the Microbial Population in Carious Lesions

**DOI:** 10.3390/ma14092387

**Published:** 2021-05-04

**Authors:** Chiara Valenti, Stefano Pagano, Silvia Bozza, Enrico Ciurnella, Giuseppe Lomurno, Benito Capobianco, Maddalena Coniglio, Stefano Cianetti, Lorella Marinucci

**Affiliations:** 1 Odontostomatological University Centre, Department of Medicine and Surgery, University of Perugia, Gambuli Square, 1, 06129 Perugia, PG, Italy; chiara.valenti@studenti.unipg.it (C.V.); benitocapobianco@libero.it (B.C.); maddalena.coniglio@studenti.unipg.it (M.C.); stefano.cianetti@unipg.it (S.C.); 2 Medical Microbiology Section, Department of Medicineand Surgery, University of Perugia, Gambuli Square, 1, 06129 Perugia, PG, Italy; silvia.bozza@unipg.it (S.B.); enrico.ciurnella@unipg.it (E.C.); 3 Oral Surgery and Ambulatory, Department of Medicine and Surgery, S. Maria della Misericordia Hospital, University of Perugia, Gambuli Square, 1, 06129 Perugia, PG, Italy; giuseppe.lomurno@unipg.it; 4 Section of Biosciences and Medical Embryology, Department of Experimental Medicineand Surgery, University of Perugia, Gambuli Square, 1, 06129 Perugia, PG, Italy; lorella.marinucci@unipg.it

**Keywords:** Er:YAG laser, conservative dentistry, oral microbiome, cariogenic species

## Abstract

The aim of this study is to investigate the Erbium:Yttrio-Aluminum-Granate (Er:YAG) laser photothermal and mechanical effects on cariogenic species concentration and on the microbial load composition of therapeutic cavities, in order to evaluate the possible micro-organisms reduction and make a comparison with manual and rotating conventional therapy (CT). A clinical trial was designed, including adults with active deep carious lesions on permanent teeth who were divided into two groups, i.e., control group and intervention group treated with CT and Er:YAG therapy, respectively. Before and after any conservative treatment, two oral samples were collected using a small sterile microbrush scrubbed within the base of the dentinal cavity tissue. The percentage of reduction and the colony-forming units (CFUs) count after Er:YAG and conventional treatments were compared for total microorganisms, including *Candida* spp., *Streptococcus* spp., and *Lactobacillus* spp. The microbial reduction varied from 90.2% to 100% and was significantly observed for total microorganisms and *Streptococcus* spp. (*p* < 0.05). The Er:YAG laser shows the potential for clinical applications, especially with paediatric and complicated patients, thanks to its minimally invasive properties and its effect on the reduction of microbial load.

## 1. Introduction

Caries is one of the most common chronic diseases with infective origin [1]. It is characterized by the destruction of hard and mineralized dental tissues due to various factors, such as bacterial metabolism, diet rich in sugars, poor oral hygiene, and the intrinsic susceptibility and socio-cultural elements of the patient (i.e., the level of education and employment status) [2]. In particular, acid metabolites released by commensal bacteria of the oral biofilm [3] leads to the demineralization of tooth surfaces and to carious cavities formation. The most recent “Specific Theory” and “Ecological Plaque Theory” of the microbial etiology of caries are related to the increased incidence of caries with the presence of specific bacterial species such as *Streptococcus mutans* and *Streptococcus sobrinus* [4], identified as the main pathogens of caries diseases [5], which lead to the dysbiosis of commensal microbiota.

The condition of dysbiosis and the selection mechanisms of particular microbial species [6,7] are correlated with different pathological states. The formation of biofilms represents the expression of microorganism virulence [8]. In the oral cavity, polymicrobial biofilms formed on dental and mucous surfaces or on implants and dental materials can cause a wide range of diseases and complications [9]. A dysbiosis of the oral biofilm can also lead to the growth of acidogenic species linked to caries such as Streptococci, Lactobacilli and Candida [6,7]. 

Conventional methods of caries removal, such as manual and mechanical rotating instruments (dentine spoon, turbine, or drill), are often perceived as painful for patients, and although the pain can be eliminated with local anesthesia, discomfort related to noise, vibration, or fear of the needle remains. In addition to the risk of removing a great deal of healthy tissue, mechanical techniques may also damage the pulp by increasing the temperature due to thermal stimulation. Different alternative treatments, such as chemo mechanical methods, ozone therapy, and laser technology, have recently been proposed to preserve the tooth structure by removing only the damaged tissue and being as conservative as possible [10,11,12]. Although there are various indications for ozone therapy use in dentistry, the risks are still too high, due to problems with the upper airways and contraindications in fragile subjects [13]. In particular, lasers have a double effect (mechanical and thermal) and potentially capable of disorganizing the microbial biofilm. This is important, because carious lesion can increase and evolve, attacking the pulp, if pathogenic microorganisms are not well eliminated in the cavity [14]. The residual microorganisms can also represent a possible cause of recurrent caries in conservative dental treatments. 

The introduction of laser therapy not only influences microbial load reduction, but also bacterial adhesion to dental surfaces [15,16]. In particular, Er:YAG therapy is a commonly used system for hard dental tissue ablation, since Erbium wavelength coincides with the absorption peak of water and hydroxyapatite, causing the removal of mineralized dental substance through micro-explosions [17]. Furthermore, compared to the healthy one, carious tissue contains even more water, so the Er:YAG laser has higher chromophores absorption on infected tissues, and it well represents a selective and conservative device for caries removal, allowing the creation of a therapeutic cavity preparation without excessive extension in healthy tissues below the lesion [18]. The Er:YAG laser combines photoablative and decontaminating properties with minimally invasive characteristics. Because of this, the benefits of treating a carious lesion with Er:YAG, compared to conventional therapy (CT), not only include less vibrations and noises for the patient, but above all bactericidal and bacteriostatic properties [19] due to high temperatures developed during irradiation. In fact, the photothermal effect developed during the ablation can be responsible for the disinfection of residual bacteria in the cavities, without causing thermal damage to the dental pulp [18]. Finally, it has to be considered that laser irradiation melts inorganic components of dentin and can also cause remineralisation [20,21,22]. In this way, the Er:YAG laser allows a seal of treated dentinal surfaces and increases the resistance to recurrent caries [23,24].

The aims of our study are to evaluate whether the dual action of the Er:YAG laser, (i.e., photothermal and mechanical) and reduces the concentration of cariogenic microbial species in therapeutic cavities, in order to avoid caries progressions and endodontic complications, and to investigate the microbial load composition of prepared cavities on permanent teeth treated with conservative Er:YAG therapy, which provides standardized “enamel removal” and “dentin removal” programs for adults with specific settings of frequency, energy, power, and air-water cooling parameters, in order to evaluate the possible microorganisms reduction and to make a comparison with the manual and rotating CT.

The null hypotheses are: (1) the Er:YAG laser is not effective in removing carious lesions and creating a therapeutic cavity;(2) there is no difference in the reduction of the microbial load between Er:YAG therapy and CT; (3) the reduction in microbial species is not qualitatively different following the two treatments.

## 2. Materials and Methods

### 2.1. Trial Design, Inclusion, and Exclusion Criteria for Participants and Sample Size

**Figure 1:**
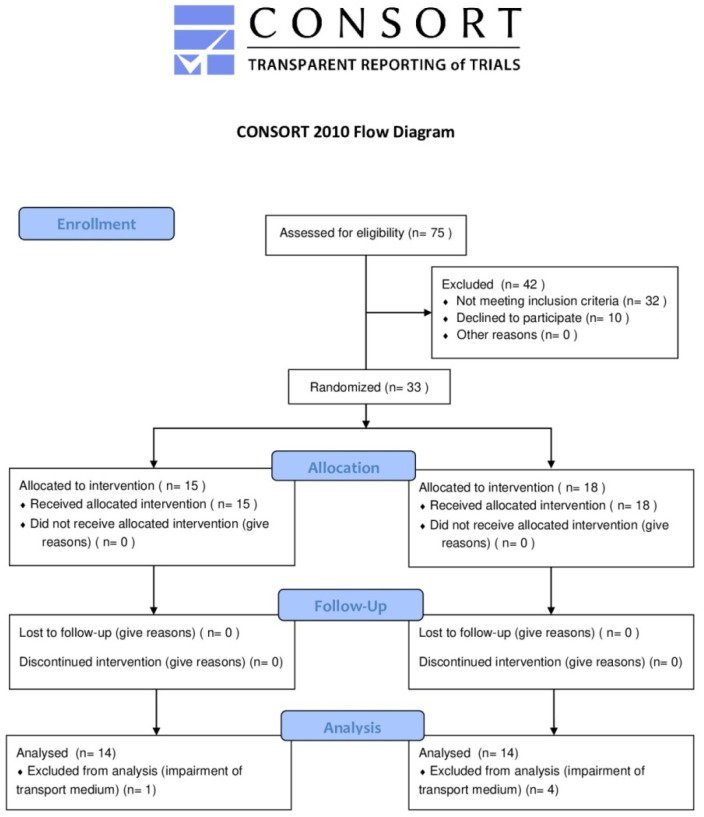
CONSORT flow diagram.

This trial was conducted in accordance with the standards of reporting trials, with CONSORT Statement [25] being the model adapted to our study design (CONSORT checklist is available as supplementary materials in Table S1). As shown in Figure 1, the CONSORT flow diagram represents in detail recruitment, allocation, follow-up, and analysis phases. 

It is a single-blind randomized controlled clinical trial (RCT). We registered this study in the international database ClinicalTrials.gov (NCT#04769882). Patients were evaluated and selected after careful oral examination in the Odontostomatological University Centre (C.O.U.) of Perugia.

Based on similar recent articles, the sample size was evaluated [26,27], and we accounted a capacity to recruit 31 patients, considering a dropout of 10% to maintain statistical significance. Patients were randomly divided with Microsoft Office Excel 2007 software into two groups: group A, control group, which involved treatment with CT; group B, intervention group, which involved treatment with Er:YAG therapy. Each treatment (group A or group B) was randomly associated with each participant on a written note inserted in numbered opaque envelopes, which were serially listed to preserve the randomization sequence.

Treatments did not involve the use of anesthesia, unless it was explicitly requested by the patient during the operation or the operator considered it necessary for the patient’s clinical conditions.

The sample consisted of adult patients of both sexes, with 18 years or older and one cavitated non-destructive carious occlusal lesion of a permanent teeth, which deepened up to the middle third of the dentin and not beyond confirmed by a radiographic exam and without pulp involvement. The exclusion criteria were as following: patients that refused to sign the informed consent document; patients with less than 18 years or children; pregnant subjects; patients with syndromes or chronic systemic diseases; patients who had used antibiotics within the previous three months or under pharmacological treatments; patients with painful symptoms of irreversible pulpitis, mobility, and destructive carious lesions extending beyond the middle third of the dentin; patients with carious lesion with the exposure of the dental pulp or periodontitis; patients with caries on deciduous teeth. After the trial started, patients who did not cooperate in the radiographical exam and/or with the treatment procedures were also excluded.

### 2.2. Data Collection

Anamnesis was performed with a standard clinical form including personal and general health data. The medical and personal information records of each patient, collected as paper documents, were transferred to a computer support in .xls file format and integrated with the results of the experimental analysis. The clinical examinations were performed by a trained operator, who underwent 12 clinical sessions of training before the procedures. Standardized periapical radiographs were performed using a radiographic Rinn positioner to avoid sprojections and evaluate the dental elements in their apico-coronal complex. Once all the data were collected and inserted in the .xls document, the statistical program Microsoft Office Excel 2007 was used. To ensure the confidentiality of the data, a serial number was assigned to each patient, and only the investigator was able to link the numbers to the corresponding person. In addition, the investigator used a computer with limited access and a password. The management of the paper material was carried out in accordance with the legislation in force on the matter.

### 2.3. Clinical Procedures and Sample Collection

The treatments were performed after isolation with a rubber dam, to achieve isolation and avoid further contamination of the operating and sampling field. The decayed tissue was removed firstly by the side walls and then by the cavity bottom. Caries removal was performed with different procedures in the two groups. The procedures for group A: manual and rotating instruments, such as a dentin spoon (ASA Dental S.p.a., Bozzano, Italy), turbine (NSK Dental ItalyS.r.l., Thiene, Italy; NAKANISHI INC., Tochigi, Japan) with diamond burs (Kerr Dental Italia S.r.l., Scafati, Italy), was used to cut the enamel and open the cavity, and drill handpiece (KaVo Dental Italia S.r.l., Genova, Italy) was used with tungsten carbide burs (Kerr Dental Italia S.r.l., Scafati, Italy) to remove the infected dentin. The procedures for group B: an Er:YAG laser (Doctor Smile, Lambda SRL, Roma, Italy) with BOOST handpieces using the“enamel removal” program for adult, “normal” modality, and selected settings (frequency: 20 Hz, energy: 400 mJ, power: 8.0 W, air: 80%, water: 60%) was adopted to cut the enamel and open the cavity, and a 90° handpiece was used with the “dentin removal” program for adult, “normal” modality, and selected settings (frequency: 20 Hz, energy: 200 mJ, power: 4.0 W, air: 90%, water: 30%) to remove the carious dentin, with tips of 800 µm in diameter and 8 mm or 12 mm in length, in relation to the depth and distance of the lesion.

After the soft carious dentin was removed, it was detected with a dental probe that dentin was hard and resistant at the base of the cavity, and tissue excavation was considered finished. This means that only the infected dentin was removed, leaving the affected dentin above the pulpal chamber. Finally, the cavity was restored with an adhesive system (Heliobond, Ivoclar Vivadent, Liechtenstein) and composite resin (Charisma, Kulzer, Mitsui Chemicals Inc., Tokyo, Japan) by incremental technique, reducing the polymerization shrinkage effects.

Dentin characteristics were classified according to Kidd et al. [28,29] in relation to the aspects of color and consistency. Dentin consistency was considered “soft” (if the probe penetrated the tissue easily), “medium” (if the probe required more pressure to penetrate), or “hard” (if the tissue was similar to the surrounding healthy dentin). Color was defined as “dark brown”, “medium brown”, or “pale”.

**Figure 2:**
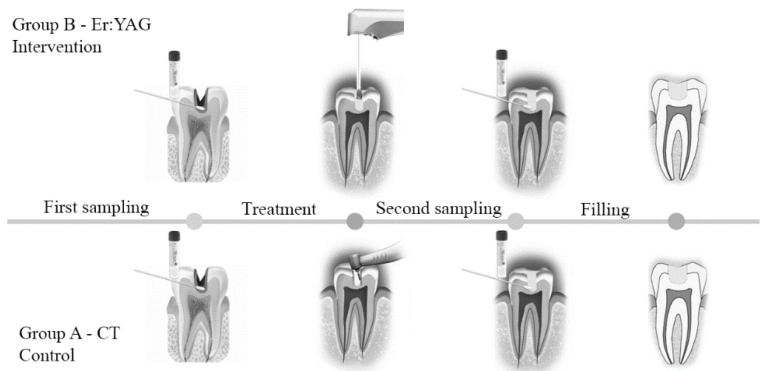
Overview of the sampling and treatment procedures for both groups:(**A**) control group; and (**B**) intervention group.

In this study, two oral samples were collected, one before and the other one after the treatments for patients of both CT and Er:YAG therapy groups. The specimen was taken using a small sterile microbrush scrubbed within the dentinal cavity bottom, in order to study the different microbial community and the percentage of microbial reduction after the treatments. This was done in order to avoid any dentin biopsy or surgical extraction of the tooth, excluding iatrogenic damages and the removal of healthy dentin together with infected, preserving a greater amount of tissue above the pulpal chamber [30]. A brief diagram in Figure 2 illustrates the treatment and sampling procedures for both the control (group A, CT) and intervention group (group B, Er:YAG therapy). The collected samples were stored in test tubes with Aimes transport medium for at least 48 h in a fridge. The microbiological analyses were conducted with the blind counting of carious dentin samples, collected before and after CT (group A) and before and after Er:YAG therapy (group B).

### 2.4. Microbiological Analysis and Procedures

**Figure 3:**
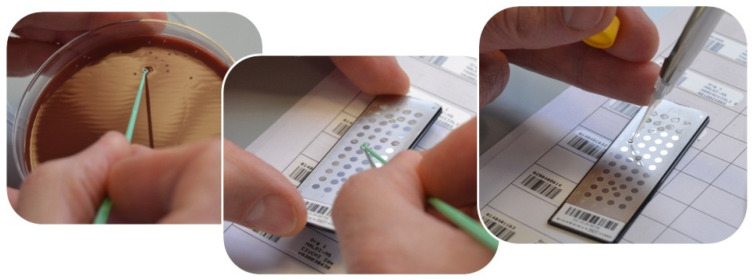
Preparation for colony typing on an MALDI TOF mass spectrometer.

Ultrapure water was added to the tubes containing dentin samples and homogenized in a tube shaker for 30 s with 3300 rpm following two cycles of ultrasonic vibration at 42,000 Hz for 8 min and agitation at 3300 rpm for 30 s to detach the microbial cells. A decimal dilution up to 1:10 was applied after performing test dilutions for microbiological analysis protocol standardization. A physiological solution with a pH value of 7.4, containing 9 g of NaCl, and 1000 mL of distilled water was used as diluent. The sample dilution technique was performed by mixing, in a sterile test tube, 1 mL of the sample and 9 mL of the diluent. The culture conditions set up for the study included the incubation of the culture media in anaerobiosis (with anaerobic jars), with an atmosphere of 80% nitrogen, 10% hydrogen, and 10% carbon dioxide, and incubation in aerobic conditions enriched with carbon dioxide at three different temperatures (i.e., 30 °C, 37 °C, and 42 °C). The samples were plated in triplicate to evaluate the microorganisms viability in different types of culture media to identify the microbial load and the colony-forming units (CFUs): Chocolate Agar with hemoglobin and yeast concentrate, for all the microorganisms; Sabouraud Agar (BD, Becton, Dickinson and Company)with chloramphenicol for fungi, such as *Candida* spp.; Columbia CNA Agar (BD, Becton, Dickinson and Company) with antimicrobial agents, such as colistin and nalidixic acid, which made it selective for gram-positive bacteria, Streptococci and Staphylococci; MacConkey Agar (BD, Becton, Dickinson and Company) for gram-negative bacteria; Shaedler Agar (BD, Becton, Dickinson and Company) for strict anaerobes; and Haemophilus Agar (from Biomèrieux Italia S.p.A.), a selective medium with factors X and V and yeast extract. Then, a pure culture was prepared by sterilely taking the microorganisms from the isolated colonies and analyzed using a mass spectrometry with MALDI TOF technology (VITEK MS Biomèrieux system, Biomèrieux Italia S.p.A.), as shown in Figure 3.

### 2.5. Data Processing and Statistical Analysis

All the data collected were tabulated and analyzed in Microsoft Office Excel 2007. Mean age and gender frequency were obtained. One-way analysis of variance (ANOVA) was performed using GraphPad Prism 8 software (Prism, CA, USA), and a significant value of *p* < 0.05 was adopted. The microbiological analysis was performed counting the dentin in the unit of CFU/mg by two blinded trained evaluators. The percentage of reduction was used to show the relative number of live microbials eliminated by both CT (group A) and Er:YAG therapy (group B). The two techniques percentage of reduction was determined and compared by calculating the difference between the dentin collected in the unit of the CFU/mg before and after the treatments. Inspired by the most recent literature on interventional in vivo studies [27], different types of microorganisms isolated in groups A and B have been considered into four “macro-groups” represented by total microorganism macro-group, *Streptococcus* spp. macro-group, *Lactobacillus* spp. macro-group, and *Candida* spp. macro-group. In order to compare control and intervention group statistical data, we analyzed the percentage of microbial load reduction, the mean CFUs per milligram and the SD of the mean CFUs per milligram.

## 3. Results

The samples collected from 33 patients in group A (*n* = 15) and group B (*n* = 18) each were subjected to microbiological analysis. During the collection, samples from 1 subject of group A were excluded due to unsuitable storage in the transport medium, and samples from 2 subjects of group B were excluded due to the contamination of the medium. A total of 56 samples, obtained from the pre- and post-treatment sampling of 28 permanent dental elements group A (*n* = 14) and group B (*n* = 14), were considered for final analysis and revealed microbial growth.

Among the samples, 43% (*n* = 12) of the patients were female, and 57% of the patients were male (*n* = 16). Analyzing the patients age, it was found that 18% were aged between 18 and 30 years, 28% were aged between 30 and 42 years, 29% were aged between 42 and 54 years, and 25% were aged between 54 and 66 years.

In relation to the dentinal characteristics and the type of treated lesions [28,29], “mid brown” dentinal dyschromia was clinically found in 43% of the lesions, “dark brown” pigmentation was clinically identified in 57% of the lesions, a “soft” dentinal consistency were clinically found in 21% of the lesions, a “medium” consistency was clinically found in 54% of the lesions, and “hard” consistency was clinically found in 25% of the carious lesions.

From the analysis carried out after the culture examination, different types of micro-organisms were identified. Table 1 shows the most representative ones.

**Figure 4:**
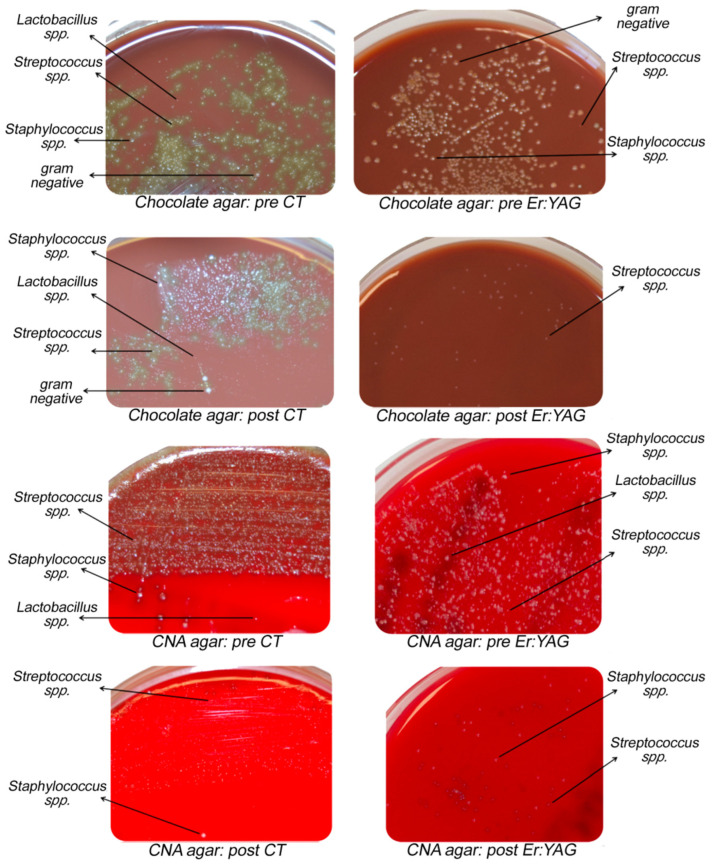
Difference in gram-positive and gram-negative colonies growth after Er:YAG treatments (**right**) vs. CT (**left**). Pre CT: pre-treatment with conventional therapy; post CT: post-treatment with conventional therapy; pre Er:YAG: pre-treatment with laser therapy; post Er:YAG: post-treatment with laser therapy.

**Figure 5:**
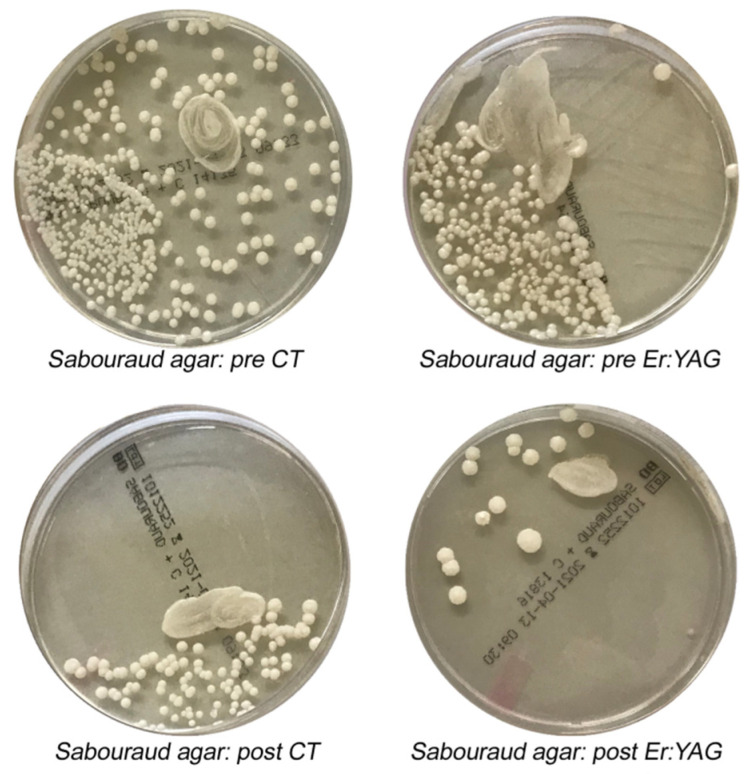
Difference in Candida albicans (*C. albicans*) colonies growth after Er:YAG treatments (**right**) vs. CT (**left**). Pre CT: pre-treatment with conventional therapy; post CT: post-treatment with conventional therapy; pre Er:YAG: pre-treatment with laser therapy; post Er:YAG: post-treatment with laser therapy.

Figure 4 and Figure 5 show the growth variations of different microbial species in various culture media before and after CT and Er:YAG treatments.

In particular, Table 2 shows the total microbial growth, with 91% of CFU reduction for samples treated with Er:YAG and 80.6% of CFU reduction for those treated with CT. The presence of bacteria belonging to the genus *Streptococcus* spp. was found in 26 permanent elements, both in the samples of the control group A (*n* = 13) and the intervention group B (*n* = 13). The percentage of reduction of the microbial load was 90.2% for the lesions treated with Er:YAG compared to a 72.4% reduction for the lesions after CT. *Lactobacillus* spp. was found in three permanent elements, both in the samples of the control group A (*n* = 2) and the intervention group B (*n* = 1). The percentage of reduction reached 100% with both Er:YAG and CT treatments. While *Candida* spp. was isolated in 13 permanent elements, both in the samples treated in the control group (*n* = 6) and in the intervention group (*n* = 8). The percentages of reduction were 94.4% in the lesions treated with Er:YAG and 89.5% after CT.

## 4. Discussion

In conservative dentistry, Er:YAG laser has been considered a valid alternative to replace traditional handpieces to remove dental carious lesions [31] due to its decontamination characteristics and high affinity for mineralized tissues and also for the advantages in terms of patient acceptability [32]. Er:YAG treatment is perceived as less painful and invasive than CT, in terms of noise and vibrations [33]. As reported by scientific research, paediatric patients have a higher compliance with laser therapy, according to its minimally invasive properties [34,35,36].

The aim of this research was to determine whether the Er:YAG is a safe and effective alternative to CT in caries removal and conservative cavity preparation, investigating its decontaminating properties and effects on the microbial load and on different types of microbial species.

Considering microbial growth in permanent teeth affected by caries, it was found that microbial reduction following Er:YAG treatments was 91% while a microbial clearance rate of 80.6% was achieved following CT.

The first null hypothesis can be rejected, as Er:YAG treatments show that cariogenic micro-organisms have been eliminated, creating a decontaminated therapeutic cavity. Schwass et al. [37] showed that irradiation with an Erbium laser leads to the complete removal of demineralized tissue and a minimally invasive preparation. As pointed out by Polizei et al. [36], Erbium lasers promote water absorption and enable dental ablative processes causing micro-explosions, water evaporation, and dental tissue expansion and expulsion. Er:YAG protocols for caries removal on permanent teeth involve pre-setting parameters to guarantee a standardized effect and a predictable cut level, avoiding operator-dependent variations in the amount of removed dental tissue [19]. To preserve the vitality of the pulp, working times are reduced, developing lowest heat doses. Er:YAG decontaminating properties allow the maximum optimization of residual tissues and a minimally invasive approach with less risk of pulpal exposure [18]; however, further investigations are needed to assess the amount of residual healthy dentine. It should be emphasized that Er:YAG lasers recognize deciduous tooth structures as an advantageous target, with lower thickness, more porous surface, and higher water and chromophores content, reaching a higher cutting power [38]. Changes in tissue mineralization could also affect different sites of the same tooth, such as recently erupted permanent teeth with thicker enamel [39]. It would be of interest to obtain more information about Er:YAG effects on different areas of the same dental elements.

In this study, no restorations were damaged, as assessed clinically and radiographically with follow-up bite-wings of both groups A and B. As shown in the literature, there are no significant differences between the integrity of the dental restorations carried out after Er:YAG therapy or CT [36]. According to the United States Public Health Service criteria (USPHS) [26] and Cvar and Ryge method [40], Er:YAG therapy and CT are comparable. This is related to Er:YAG ability to increase enamel resistance to demineralization, prevent secondary caries or infiltration by reducing acid dissolution [41,42,43], induce physical changes in tooth substrate (melting and recrystallisation) [44], create a porous and rough surface to provide micro-mechanical bonding for enamel-dental adhesive systems [45] and achieve a marginal seal in the interface zone of the restoration, reducing the availability of exogenous nutrients for residual microorganisms in the cavities [46]. The reduced acid solubility of dental enamel after laser irradiation and heating could be related to the expansion of free water causing tooth substrate variations or to carbonate release. Cecchini et al. [47] demonstrated that lower irradiation energies can increase enamel solubility without causing alterations in tooth structure (micro-cracks). In addition, how the operative protocol provides for the use of a handpiece in “non-contact” mode by obtaining less overheating of the tissuesshould be considered. Moreover, the Er:YAG laser produces a partial denaturation of collagen in the organic matrix of dentin, causing collagen fibrils to fuse. These structural changes are more concentrated in the surface areas and form a laser-modified layer. Although collagen degradation could lead to a decrease in the adhesion strength of laser-irradiated dentin, which could limit the diffusion of resin into the dentinal intertubular substrate, acid etching, and rinsing with water can remove the modified layer [48]. In addition, it should be considered that irradiation with the Er:YAG laser opens dentinal tubules and could facilitate the formation of a hybrid layer, which the primer and adhesive can penetrate better given the presence of the smear layer.

The second null hypothesis can be rejected, because the Er:YAG laser provides a significant reduction in microbial load, testifying good anti-microbial properties. In terms of average CFUs reduction, the Er:YAG laser proves to be effective compared to CT, in particular for active and demineralized carious lesions, with 91% and 80.6%reductions, respectively. Our results are in agreement with the literature [49]. In fact, the increase in temperature induced by laser ablation influences micro-organisms viability, modifying their cellular structure and eliminating bacteria involved in the carious process. Even if the Er:YAG laser does not guarantee sterilizing power, the residual micro-organisms appear to be irrelevant for the progress of recurrent or secondary caries underneath the restorations [26].

The third null hypothesis can be rejected, as Er:YAG anti-microbial effects are more advantageous not only for the total load of microorganisms, but in particular for streptococcal population. *Streptococcus* spp. rate of reduction was 90.2% after Er:YAG treatments compared to a 72.4% reduction after CT. Regarding the genus *Lactobacillus* spp., the percentage of reduction reached 100% with both the Er:YAG therapy and CT. While *Candida* spp. had a 94.4% reduction in lesions treated with the Er:YAG therapy and a 89.5% reduction after CT. Our results are in agreement with the scientific literature. Er:YAG protocols are effective in reducing the total microbial load, mainly characterized by genera *Streptococcus*, *Lactobacillus*, and *Candida*, which represent the microbial macrogroups most frequently investigated [26,27].

We highlighted some critical points, according with other published works [26,35,50], in particular challenges in assuming a correct working position to access the carious lesion by the presence of an fiber-optical transport system and difficulty in maintaining an accurate focus and the localization of acutting point. Furthermore, it should be considered that laser instrumentation is still expensive compared to the traditional one, even if both the technological progress and the possibility of implementing preventive, minimally invasive and early treatments, which can avoid complex dental therapies, are able to reduce the costs of these tools. An important limit of this research concerns the methodology selected for microbial culture, which is limited exclusively to sowing in standard culture media. Since the microbial species capable of growing in such conditions are limited, future research using more advanced technologies, such as NGS 16s rRNA sequencing, will be necessary to avoid losing valuable data.

## 5. Conclusions

Considering the limitations of the study, we can conclude that:

(1) Er:YAG laser irradiation allowed the complete minimally invasive removal of the carious process and the creation of a conservative therapeutic cavity preparation, increasing resistance to acid dissolution, modifying enamel surface ultrastructure, and creating a micro-retentive surface for adhesive restorations.
(2) There were significant differences in terms of the reduction of the total microbial load following treatment between Er:YAG therapy and CT.
(3) The reductions of the microbial load following the two treatments were different in various microbial populations. For the genus *Streptococcus* spp., the reduction rates with laser therapy were significant.

### Future Perspectives

Considering further developments of this technology, Er:YAG therapy would play a key role in treating uncooperative or odontophobic patients and in pediatric dentistry, intercepting early childhood caries. We could hypothesize that at higher wavelengths, with shorter pulsation times and repeated administrations, the ablative efficiency would be more optimized by developing minimal residual energy, with less heat production affecting the pulp by further reducing the risk of necrosis and thermal damage, especially in the presence of exposed dentinal tubules or microcracks. It would also be interesting to consider for future studies to compare the effect on active and inactive carious lesions with different microbial patterns, since the Er:YAG laser acts selectively on dental chromophores like hydroxyapatite and water, which are more representative in active carious lesions. We can consider this research as a preliminary study that could be used as a guide for future investigations about the effect of the Er:YAG laser on oral micro-organisms and dental tissues. However, for future studies, long-terms follow-up and microbiological analyses with more advanced technologies (NGS sequencing) would be needed to confirm the exclusive use of the Er:YAG laser as a substitute for CT in clinical dental practice.

## Figures and Tables

**Table 1:** Most represented microbial species and their phyla, divided into gram-positive and gram-negative.

Microrganism	Phylum	Gram-Positive	Gram-Negative
Staphylococcus aureus	Firmicutes	x	
Staphylococcus epidermidis	Firmicutes	x	
Streptococcus oralis	Firmicutes	x	
Streptococcus mutans	Firmicutes	x	
Streptococcus salivarius	Firmicutes	x	
Haemophilus parainfluenzae	Proteobacteria		x
Neisseria flava	Proteobacteria		x
Neisseria sicca	Proteobacteria		x
Candida albicans			
Lactobacillus casei	Firmicutes	x	
Lactobacillus collinoides	Firmicutes	x	
Lactobacillus salivarius	Firmicutes	x	
Prevotella oralis	Bacteroidetes		x
Prevotella denticola	Bacteroidetes		x
Streptococcus anginosus	Firmicutes	x	
Streptococcus cristatus	Firmicutes	x	
Streptococcus gordonii	Firmicutes	x	
Streptococcus mitis	Firmicutes	x	
Streptococcus milleri	Firmicutes	x	
Streptococcus pyogenes	Firmicutes	x	
Streptococcus sanguinis	Firmicutes	x	
Streptococcus parasanguinis	Firmicutes	x	
Actinobacillus hominis	Proteobacteria		x
Actinomyces odontolyticus	Actinobacteria	x	
Clostridium perfringens	Firmicutes	x	
Corynebacterium propinquum	Actinobacteria	x	
Escherichia coli	Proteobacteria		x
Fusobacterium nucleatum	Fusobacteria		x
Neisseria mucosa	Proteobacteria		x
Neisseria perflava	Proteobacteria		x
Staphylococcus hominis	Firmicutes	x	
Staphylococcus warneri	Firmicutes	x	

**Table 2:** This table report the percentages of colony-forming units (CFUs) reduction (%) and CFUs performed in triplicate (mean  ± SD) for each micro-organisms macro-group considered for both the intervention group B (Er:YAG) and vs. the control group A (CT) samples. A comparison of the CFUs percentages of reduction and mean CFUs (SD) for post- and pre-treatment between group B (Er:YAG) and group A (CT) was performed for each of the four selected macro-groups of micro-organisms. * *p* < 0.05. The mean CFU was only performed in the culture media in which there was the growth of the genus under investigation.

Sample Collection	Percentage of CFUs Reduction after Treatments (%)		Mean CFUs (SD)			
	Group A	Group B	CT		Er:YAG	
			Pre	Post	Pre	Post
Total microrganisms	80.6%	91% *	3.69 × 10^4^ (4.56 × 10^4^)	7.78 × 10^3^ (2.25 × 10^4^)	5.15 × 10^4^ (4.77 × 10^4^)	5.52 × 10^3^ (1.44 × 10^4^)
*Streptococcus* spp.	72.4%	90.2% *	6.04 × 10^4^ (4.64 × 10^4^)	1.84 × 10^4^ (3.54 × 10^4^)	8.01 × 10^4^ (3.96 × 10^4^)	9.40 × 10^3^ (2.18 × 10^4^)
*Lactobacillus* spp.	100%	100%	5.50 × 10^3^ (4.50 × 10^3^)	0 (0)	1 × 10^3^ (0)	0 (0)
*Candida* spp.	89.5%	94.4%	3.17 × 10^3^ (3.39 × 10^3^)	5.00 × 10^2^ (5.00 × 10^2^)	3.06 × 10^4^ (4.40 × 10^4^)	2.00 × 10^3^ (3.61 × 10^3^)
*p* value	0.05		0.05			

## Data Availability

Not applicable.

## Supplementary Materials

The following are available online at https://www.mdpi.com/article/10.3390/ma14092387/s1, Table S1: CONSORT checklist.
